# Genotyping and Molecular Characterization of VP6 and NSP4 Genes of Unusual Rotavirus Group A Isolated from Children with Acute Gastroenteritis

**DOI:** 10.1155/2024/3263228

**Published:** 2024-07-04

**Authors:** Charilaos Dellis, Elizabeth-Barbara Tatsi, Dimitra-Maria Koukou, Filippos Filippatos, Evangelia-Eirini Vetouli, Emmanouil Zoumakis, Athanasios Michos, Vasiliki Syriopoulou

**Affiliations:** ^1^ First Department of Pediatrics Infectious Diseases and Chemotherapy Research Laboratory Medical School National and Kapodistrian University of Athens “Aghia Sophia” Children's Hospital, Athens, Greece; ^2^ University Research Institute of Maternal and Child Health and Precision Medicine, Athens, Greece; ^3^ Department of Microbiology “P. & A. Kyriakou” Children's Hospital, Athens, Greece

## Abstract

Group A rotavirus (RVA), which causes acute gastroenteritis (AGE) in children worldwide, is categorized mainly based on VP7 (genotype G) and VP4 (genotype P) genes. Genotypes that circulate at <1% are considered unusual. Important genes also include VP6 (genotype I) and NSP4 (genotype E). VP6 establishes the group and affects immunogenicity, while NSP4, as an enterotoxin, is responsible for the clinical symptoms. The aim of this study was to genotype the VP6 and NSP4 genes and molecularly characterize the NSP4 and VP6 genes of unusual RVA. Unusual RVA strains extracted from fecal samples of children ≤16 years with AGE were genotyped in VP6 and NSP4 genes with Sanger sequencing. In a 15-year period (2007–2021), 54.8% (34/62) of unusual RVA were successfully I and E genotyped. Three different I and E genotypes were identified; I2 (73.5%, 25/34) and E2 (35.3%, 12/34) were the most common. E3 genotype was detected from 2017 onwards. The uncommon combination of I2-E3 was found in 26.5% (9/34) of the strains and G3-P[9]-I2-E3 remained the most frequent G-P-I-E combination (20.6%, 7/34). Children infected with RVA E2 strains had a statistically higher frequency of dehydration (50%) than those infected with RVA E3 strains (*p* = 0.019). Multiple substitutions were detected in NSP4, but their functional effect remains unknown. The result indicates the genetic diversity of RVA strains. Continuous surveillance of the RVA based on the whole genome will provide better knowledge of its evolution.

## 1. Introduction

Group A rotavirus (RVA) is one of the most common etiological agents of severe acute gastroenteritis (AGE) in infants and young children, especially in developing countries. Children with RVA AGE can present severe dehydration that can even lead to death if left untreated. RVA is responsible for more than 100,000 deaths each year worldwide [1].

RVA is a nonenveloped, icosahedral, double-stranded RNA virus (dsRNA) and is a member of the *Reoviridae* family. Its genome consists of 11 linear dsRNA segments which encode six structural viral proteins (VP1-VP4, VP6, and VP7) and six nonstructural viral proteins (NSP1-NSP6) [2].

The viral particles consist of a triple-layered capsid. The outer capsid consists of the glycoprotein VP7 and the spike protease-sensitive protein VP4. The middle layer consists of VP6 and the core layer comprises of VP2 which encapsulates genomic RNA and viral replication components [2]. The abundant VP6 protein is commonly used for the detection and classification of rotaviruses. Currently, nine rotavirus species (A-D and F-J) have been recognized by the International Committee on Taxonomy of Viruses (ICTV); however, two additional putative rotavirus species K and L have also been proposed [3]. Only species A, B, C, and H can infect humans, including animal-human transmission [4].

Group A rotaviruses are further classified based on the outer layer proteins VP7 and VP4 in G and P genotypes, respectively. Although many different G and P types have been identified so far, the most common circulating genotypes are G1-P[8], G2-P[4], G3-P[8], G4-P[8], G9-P[8], and G12-P[8] [5, 6]. Genotyping can also be applied to the whole virus genome, Gx-P[x]-Ix-Rx-Cx-Mx-Ax-Nx-Tx-Ex-Hx, where “x” indicates the number of the corresponding genotype of the genes VP7-VP4-VP6-VP1-VP2-VP3-NSP1-NSP2-NSP3-NSP4-NSP5 [7].

The gene NSP4, except for the full-length protein, encodes a toxic peptide (114–135 amino acids) and both act as enterotoxins that can stimulate Ca^2+^ release from the endoplasmic reticulum into the cytoplasm [8, 9]. NSP4 enterotoxin activity aggravates the symptoms of gastroenteritis, particularly diarrhoea and vomiting [10, 11].

Since 2006, several RVA vaccines have been released worldwide. The most widely used are the two-dose monovalent vaccine Rotarix (GlaxoSmithKline Biologicals, Belgium) and the three-dose pentavalent vaccine RotaTeq (Merck, United States), which cover the most common G and P genotypes [12, 13]. After their release, notable changes in genotype distribution have been described worldwide, such as the increase in unusual G and P genotypes [2, 14]. The aim of this study was the genotyping and molecular characterization of VP6 and NSP4 genes of previously described [15] unusual G and P RVA strains isolated from children ≤16 years with AGE to find out the virulence of these strains and their possible coverage by circulating vaccines against rotavirus.

## 2. Materials and Methods

### 2.1. Study Design

This is a retrospective study involving RVA-positive fecal samples with previously described unusual G (G6, G8, and G10) and/or P (P[6], P[9], P[10], P[11], and P[14]) genotypes collected from children ≤16 years hospitalized with AGE [15]. Children were admitted to “Aghia Sophia” Children's Hospital with AGE symptoms during 2007-2021. Fecal samples were collected within the first two days after admission and the RVA antigen detection test (Rota-Adeno Combo Rapid kit, Hangzhou Alltest Biotech Co., China) was performed during the first eight hours of sample collection. Positive RVA fecal samples were stored properly at 2–8°C and were genotyped within five days [15].

The G and P genotyping of the isolated strains was performed as previously described [15] using agarose gel electrophoresis and sanger sequencing, as part of the annual surveillance of circulating RVA, in the Infectious Disease and Chemotherapy Research Laboratory of National and Kapodistrian University of Athens, Greece. As unusual RVA genotypes were defined as the G and P genotypes that circulate in a very low percentage (≤1%) in the population and/or have an animal origin, the unusual RVA genotypes are not included in the vaccines, and hence their coverage remains unknown. Besides, their virulence is also important to be examined. In the present study, these strains were further genotyped in VP6 (I genotype) and NSP4 (E genotype) genes.

Demographic and epidemiological data such as age, gender, residence, and RVA vaccination status were also collected from children infected with an unusual RVA genotype. Clinical symptoms (diarrhoea, vomiting, fever, and dehydration), coinfection and laboratory data (measurements in blood of potassium (K^+^), sodium (Na^+^), calcium (Ca^2+^), chlorine (Cl^−^), C-reactive protein (CRP), urea, creatinine, white blood cells (WBC), polymorphonuclear leukocytes, and lymphocytes) were also recorded. Vesikari severity score was also estimated [16].

The Scientific and Bioethics Committee of “Aghia Sophia” Children's Hospital approved this study (no. 6261).

### 2.2. VP7 and VP4 Genotyping

Nucleic acid extraction and reverse transcription (RT) were performed as previously described [15] within the first five days after sample collection. In brief, positive RVA samples were isolated using MagNA Pure Compact Nucleic Acid Isolation Kit I (Roche Diagnostics, Basel, Switzerland) on a MagNa Pure Compact instrument following the manufacturer's instructions. The extracted RNA was reverse transcribed using the Transcriptor first-strand cDNA synthesis kit (Roche Diagnostics, Basel, Switzerland). VP7 and VP4 genes were amplified using specific primers according to the European Rotavirus Network Detection and Typing Methods (European Rotavirus Network 2009). Sanger sequencing was performed using the BigDye Terminator v3.1 cycle sequencer kit on an Applied Biosystems 3500 genetic analyzer (Applied Biosystems, Waltham, MA, USA) and the BLAST bioinformatics tool (https://blast.ncbi.nlm.nih.gov/Blast.cgi) was used for G and P genotyping.

### 2.3. Amplification of VP6 and NSP4 Genes

PCR amplification was performed using the GoTaq DNA polymerase (Promega, Madison, WI, USA) and the primers F: 5′-GAC GGV GCR ACT ACA TGG T-3′ plus R: 5′-GTC CAA TTC ATN CCT GGT G-3′ for the VP6 gene and F: 5′-GGC TTT TAA AAG TTC TGT TCC GAG-3′ plus R: 5′-GTC ACA YTA AGA CCR TTC CTT CCA T-3′ for the NSP4 gene [17, 18]. The PCR amplification was carried out with an initial denaturation at 94°C for 2 minutes (min), followed by 40 cycles of denaturation for 1 min at 94°C, annealing for 1 min at 55°C for the VP6 gene and 48°C for the NSP4 gene, extension for 1 min at 72°C, and final extension for 10 min at 72°C. The amplification products were analyzed by 2% agarose gel electrophoresis using a 50 bp DNA ladder (N3236S; New England Biolabs, Massachusetts, USA) and ethidium bromide staining. If PCR products did not exist in electrophoresis gel, the genotype was characterized as unidentified (UD).

### 2.4. Sequencing and Phylogenetic Analysis

The I and E genotypes were determined by performing Sanger sequencing with the BigDye Terminator v3.1 cycle sequencer kit on an Applied Biosystems 3500 genetic analyzer (Applied Biosystems, Waltham, MA, USA) and by using the BLAST bioinformatics tool (https://blast.ncbi.nlm.nih.gov/Blast.cgi).

The VP6 and NSP4 sequences were compared with reference strains from the Wa, DS-1, and AU-1 constellations depending on the I and E genotypes to detect substitutions. The reference strains K02086.1 (Wa), DQ870507.1 (DS-1), and DQ490538.1 (AU-1) were used for the molecular characterization of the VP6 sequence, and the AF093199.1 (Wa), EF672582.1 (DS-1), and D89873.1 (AU-1) were used for the characterization of the NSP4 sequence.

Phylogenetic evolutionary analysis was performed on VP6 and NSP4 genes using the MEGA 11 software (Molecular Evolutionary Genetics Analysis; https://www.megasoftware.net). Multiple sequence alignment was performed using MUSCLE software (Multiple Sequence Comparison by Log-Expectation). The nucleotide substitution model was selected based on the BIC (Bayesian information criterion) scores using MEGA11. The model used in this study was the Tamura 3-parameter (T92) and the rate variation model, which allows some sites to be evolutionarily invariant (+I). The evolutionary tree was constructed using the maximum likelihood method and bootstrap resampling with 1000 replicates.

### 2.5. Statistical Analysis

Data statistical analysis was carried out using SPSS software (IBM Statistical Package for Social Sciences for Windows, Version 25.0., IBM Corp, Armonk, NY). A *p* value of ≤0.05 was considered statistically significant. Differences among variables were assessed using Pearson's chi-square (*χ*^2^ test). Fisher's exact test was used to analyze two or more categories or when the criteria for the *χ*^2^ test were not met.

### 2.6. Nucleotide Sequence Accession Numbers

The nucleotide sequences of this study were deposited in GenBank database (https://www.ncbi.nlm.nih.gov/genbank/) with accession numbers OM281957-59, OM287400, OM303085, OM303088, OM333185, OM333186, OM323986, OM461377, OM461378, OM972707-OM972710, ON004913, ON009342, ON156796, ON156797, ON185611-18, ON206978, ON206980-83, ON971933, and ON971934 for the VP6 gene and OM281953, OM281956, OM283121-26, OM287398, OM287399, OM362404, OM948988-91, ON004914, ON156785-93, ON564370, ON564371, and ON971935 for the NSP4 gene (Supplementary Table 1).

## 3. Results

### 3.1. I and E Genotyping and Genetic Linkage with G and P Genotypes

From 2007 to 2021, 54.8% (34/62) of the unusual RVA strains consisting of 5.9% (2/34) of unusual G (G8 and G10), 64.7% (22/34) of unusual P (P[6], P[9], P[10], and P[11]), and 29.4% (10/34) of unusual G and P (G6-P[9], G6-P[14], and G8-P[14]) were successfully genotyped for I and E.

**Figure 1:**
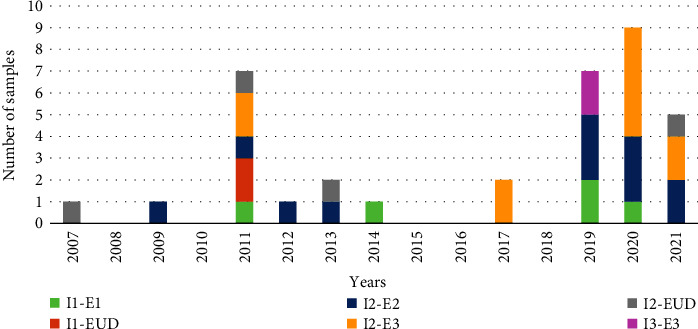
I-E genotype annual distribution of 34 unusual rotavirus group A strains isolated from children aged ≤16 years hospitalized with acute gastroenteritis during 2007–2021. EUD, unidentified E genotype.

Both I and E genotypes of three different types were identified: I1 (7/34, 20.6%), I2 (25/34, 73.5%), I3 (2/34, 5.9%) and E1 (5/34, 14.7%), E2 (12/34, 35.3%), E3 (11/34, 32.4%). The most common genotypes were I2 and E2. The E3 genotype was first detected in samples in 2017 and was the second most common E genotype (Figure 1).

E3 was detected in strains with P[9] genotype combined with G3 (*n* = 8), G4 (*n* = 1), G6 (*n* = 1), and G9 (*n* = 1) genotypes (Table 1).

Six (6/34, 17.6%) samples were not successfully genotyped in the NSP4 gene, and they were characterized as EUD (unidentified E genotype). These RVA strains were the following: G2-P[6]-I2-EUD (*n* = 1), G6-P[9]-I2-EUD (*n* = 2), G8-P[8]-I1-EUD (*n* = 1), G8-P[14]-I2-EUD (*n* = 1), and G12-P[6]-I1-EUD (*n* = 1) (Table 1).

The most frequent combinations of G-P-I-E were G3-P[9]-I2-E3 and G8-P[14]-I2-E2 accounting for 20.6% (7/34) and 11.8% (4/34) of the samples, respectively (Table 1).

### 3.2. Association of I and E Genotypes with Patient Characteristics

Statistical analysis of demographic, clinical, and laboratory data from children depending on RVA I genotype showed no significant association (Supplementary Tables 2 and 3). The corresponding analysis on E genotypes showed that children infected with E2 RVA strains had a higher relative frequency of dehydration (6/12, 50%) than those with the E3 genotype (0/9, 0%) (*p*=0.019). Vesikari severity score was estimated for 30/34 patients and is shown in Supplementary Table 1. Symptoms of severe gastroenteritis were seen in 16/30 (53.3%) children.

### 3.3. Molecular Characterization and Phylogenetic Analysis of VP6

Molecular characterization was performed in the VP6 gene fragment, which encodes the protein amino acids (aa) 243–368, after comparison with the reference strains. This comparison showed four homozygous missense substitutions (V252I (*n* = 7/7), I281V (*n* = 3/7), A287T (*n* = 7/7), and L291S (*n* = 7/7)) in strains carrying the I1 genotype, four (V281I (*n* = 19/25), S303A (*n* = 25/25), M342L (*n* = 1/25), and V349I (*n* = 1/25)) in strains carrying the I2 genotype, and one (V330I) in both strains carrying the I3 genotype. However, none of these substitutions was *novel* after comparison of VP6 sequences with the 100 most similar strains using BLAST.

In the sequenced fragment of the VP6 gene, a part of the antigenic region III (aa 208–274) was included. Genetic analysis revealed the existence of three already known substitutions; the homozygous Y248F carried by all I1, Wa, and Rotarix strains, homozygous V252I carried by all I1 strains, and the homozygous I253V carried by I3 (*n* = 1) and AU-1 strains.

**Figure 2:**
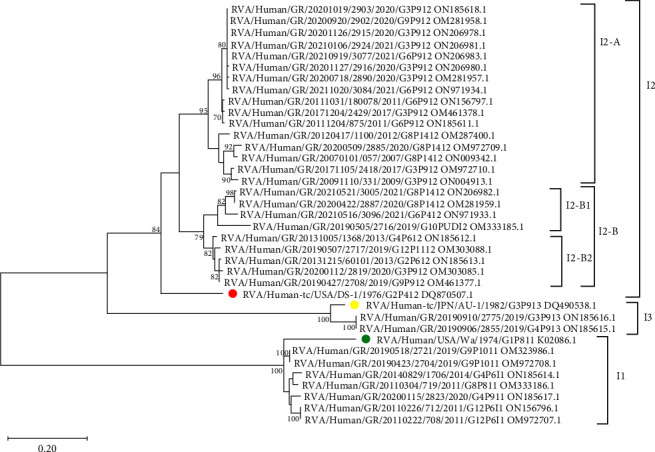
Phylogenetic tree of VP6 gene of unusual rotavirus group A strains (*n* = 34) circulating in Greece between 2007 and 2021. Reference strains are indicated by a colored circle. The tree was constructed using the maximum likelihood method and T92 + G + I model [19]. Bootstrap values (1000 replicates) above 70% are shown. The scale bar indicates the branch length for 0.20 substitutions per nucleotide position. Evolutionary analysis was conducted with MEGA 11 software [20].

Phylogenetic analysis of the VP6 gene in 34 unusual RVA strains revealed three distinct groups corresponding to I1, I2, and I3 genotypes with 100% reliability for the I1 and I3 groups and 84% reliability for the I2 group. Among the unusual RVA strains carrying the I2 genotype, two distinct clades (I2-A and I2-B) were identified with 95% and 79% reliability, respectively (Figure 2).

The division of these clades is based on the 9 synonymous substitutions (L265L/c.793T > C, N266N/c.798T > C, Y273Y/c.819T > C, T287T/c.861T > A, L294L/c.882A > G, V304V/c.912G > A, L324L/c.972A > G/T, A344A/c.1032T > A, and T347T/c.1041G > A). Clade I2-B is also divided into two subclades (I2-B1 and I2-B2). This separation also occurred due to the substitutions of the missense V281I/c.841G > A (carried by 5 strains) and four synonymous (N25N/c.75T > C, A275A/c.825A > T, Τ323Τ/c.969G > A, and L324L/c.972T > G) substitutions.

### 3.4. Molecular Characterization and Phylogenetic Analysis of NSP4

**Figure 3:**
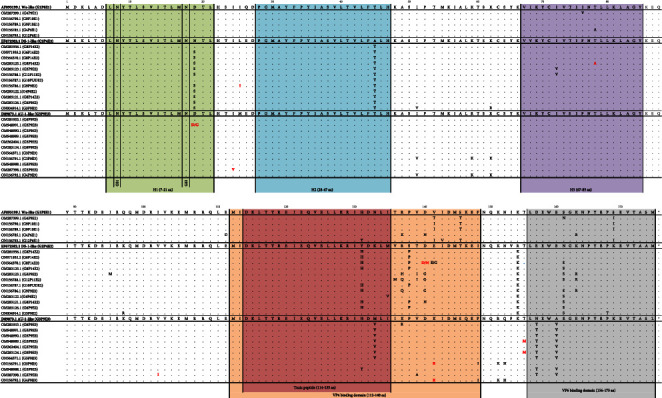
Multiple sequence alignment of the NSP4 proteins (*n* = 28) of unusual human rotavirus group A strains. E1, E2, and E3 strains were compared with Wa (AF093199.1), DS-1 (EF672582.1), and AU-1 (D89873.1), respectively. H1, hydrophobic domain 1; H2, hydrophobic domain 2; H3, hydrophobic domain 3; GS1, glycosylation site 1; GS2, glycosylation site 2.

Molecular characterization was performed *ο*n the whole NSP4 gene. Through this comparison, 13 homozygous missense substitutions were found in strains carrying the E1 genotype, 21 homozygous and two heterozygous missense substitutions in strains carrying the E2 genotype, and 16 homozygous and one heterozygous missense substitution in strains carrying the E3 genotype (Figure 3). Most of these substitutions (*n* = 23) were located in the VP4-binding region (aa 112–148).

The NSP4 gene sequences were compared with the 100 most similar strains using BLAST, and eight possibly *novel* substitutions were identified. These *novel* substitutions were the D140N in one E1 strain; the L25I (*n* = 1), T78A (*n* = 1), and D140N (*n* = 2) in four E2 strains; and the D19G (*n* = 1), I24V (*n* = 1), V102I (*n* = 1), K141R (*n* = 2), and T155M (*n* = 2) in six E3 strains. Four of these substitutions were located within significant domains of NSP4. Specifically, D19G and T78A were in the conserved hydrophobic domains 1 and 3 (H1 and H3), respectively, and D140N and K141R were located in the VP4-binding domain.

In the toxic peptide region, three already known homozygous substitutions were detected. The H131Y was found in 1/5 (20.0%) E1 strain, in 4/12 E2 strains (33.3%), and in 1/11 (9.1%) E3 strain. The M133V was found in 7/11 (63.6%) E3 strains and the M135V was detected in 1/12 (8.3%) E2 strain (Figure 3). The significant substitutions observed in the NSP4 gene are shown in Table 2.

**Figure 4:**
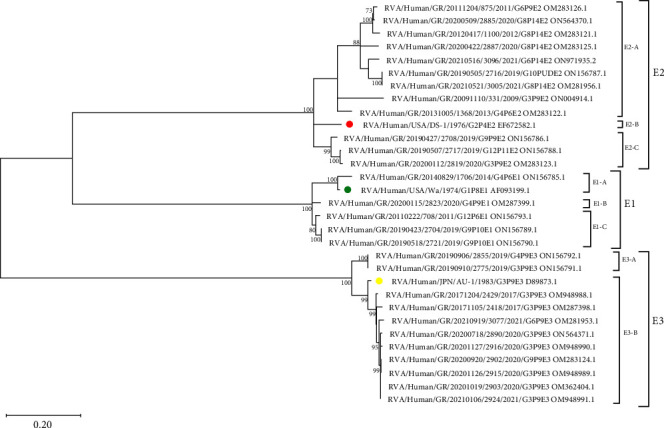
Phylogenetic tree of NSP4 gene of unusual RVA strains circulating in Greece between 2007 and 2021. Reference strains are indicated by a colored circle. The tree was constructed using the maximum likelihood method and T92 + I model [19]. Bootstrap values (1000 replicates) above 70% are shown. The scale bar indicates the branch length for 0.20 substitutions per nucleotide position. Evolutionary analysis was conducted with MEGA 11 software [20].

Phylogenetic analysis of the NSP4 gene in 28 unusual RVA strains revealed three distinct groups corresponding to E1, E2, and E3 genotypes with 100% reliability. Among the unusual RVA strains carrying the E2 genotype, three distinct clades (E2-A, E2-B, and E2-C) were identified (Figure 4).

The division of the E2-A clade from E2-B and E2-C is based on four synonymous substitutions (L21L/c.63A > G, I56I/c.168A > T, L116L/c.346C > T, and V124V/c.372A > T). The E2-A clade differentiated from the E2-B clade due to one missense (A45T/c.133G > A) and additional five synonymous (N18N/c.54T > C, Q109Q/c.327A > G, L110/c.330A > G, I130I/c.390C > T, and S138S/c.414G > A) substitutions and from the E2-C clade due to one missense (G140D/c.419G > A) and another four synonymous (P34P/c.102C > T, E125E/c.375G > A, I130I/c.390A > T, and P168P/c.504G > A) substitutions. The E2-B clade differed and separated from the E2-C clade due to two missense (A45T/c.133G > A and G140D/c.419G > A) and nine synonymous substitutions.

Unusual strains carrying the E1 and E3 genotypes were also divided into 3 (E1-A, E1-B, and E1-C) and 2 (E3-A and E3-B) distinct clades, respectively (Figure 4). Separation between the E1-A and E1-B strains occurred due to three missense (I141V/c.421A > G, T145S/c.433A > T, and I169S/c.505_506AT > TC) and 22 synonymous substitutions. The E1-C clade differed from both E1-A and E1-B clades due to two missense (I76V/c.226A > G and S161N/c.482G > A) and three synonymous (K3K/c.9G > A, L82L/c.244_246TTG > CTA, and P138P/c.414A > G) substitutions. Furthermore, the E1-C clade differed from the E1-A clade in three missense (V141T/c.421_422GT > AC, S145T/c.433T > A, and S169I/c.505_506TC > AT) and 21 synonymous substitutions and from the E1-B clade in one missense (I141T/c.422T > C) and seven synonymous substitutions. The division among the E3 cluster appeared due to six missense (I51V/c.151A > G, R59K/c.176G > A, R141K/c.422G > A, F148I/c.442T > A, R151K/c.452G > A, and Q152H/c.456A > C) and 25 synonymous substitutions (Figure 4).

## 4. Discussion

There are limited studies that investigate the molecular characterization of VP6 and NSP4 genes in human RVA strains worldwide as the interest has mainly focused on G and P distribution. This 15-year study focusses on the genotyping and molecular characterization of VP6 and NSP4 genes of unusual G and P RVA strains isolated from children hospitalized with AGE.

Genotyping revealed three different I (I1, I2, and I3) and E (E1, E2, and E3) genotypes in unusual RVA strains, I2 and E2 being the most common. According to the rotavirus classification working group, 32 I and E genotypes are known so far, with I1, I2 and E1, E2 being the most commonly detected genotypes among humans [8, 21–23]. I1-E1 are strongly associated with G1/G3/G4/G5/G9-P[8] and follow the Wa-like genotype constellation, I2-E2 are associated with G2-P[4] typical of the DS-1-like genotype constellation, and I3-E3 are associated with G3-P[9] typical of the AU-1-like constellation [22, 24].

Similarly, in a 10-year study (1996–2006) conducted in Brazil, which included both common and unusual strains, they found the same three I and E genotypes [24]. In their study, the most prevalent I and E genotypes were I1 (82.7%) and E1 (81.5%), respectively. However, among strains with an unusual G (G6, G8, and G10) and/or P (P[6], P[9], P[10], P[11], and P[14]) genotype, I2 and E2 were the most prevalent I and E genotypes (*n* = 7/13) [24], as in the present study. In other epidemiological studies such as a 4-year study conducted in the Democratic Republic of Congo, although the number of unusual G and/or P strains recorded was substantial, only two I (I1 and I2) and E (E1 and E2) genotypes were recorded [25].

In this study, I3 and E3 were detected in 2019 and 2017 onwards, respectively. Strains carrying the E3 genotype showed a significant increase between 2019 and 2021 during the COVID-19 pandemic period and they were mostly found in combination with G3-P[9]-I2 (*n* = 5/21, 23.8%). G3-P[9]-I2-E3 was the most prevalent G-P-I-E genotype combination throughout this study (*n* = 7/34, 20.6%). The increase in E3 was observed in the same period with the increase of P[9] strains in Greece, as recorded by Tatsi et al. [15]. The first record of the G3-P[9]-I2-E3 genotype in humans was in 2012 in Korea, where it was isolated from a 9-year-old female [26]. However, a similar strain (G3-P[9]-I2-R2-C2-M2-A3-N2-T3-E3-H3) was recently identified in 2021 in Thailand, which originated from a feline with diarrhoea [27].

The rare combination of G3-P[9]-I2-E3 that was detected in this study is possibly derived from a reassortment event, but further investigation should be performed. Reassortment is common among RVs and is a crucial mechanism for the evolution of the virus. Molecular characterization of multiple RVA genes is important, as it may contribute to detecting strains that do not fit into any of the major constellations (Wa, DS-1, and AU-1) and are probably products of reassortment events. Furthermore, this finding supports that VP6 and NSP4 can segregate independently, contradicting a study in 2003 that reported a genetic linkage among these two proteins in common, unusual, and reassortant human strains [22]. Similarly to our observation, many studies reported such reassortment events at VP6 and NSP4, but at a lower rate. In an 11-year study (1996–2006) in Brazil, the I1-E2 unusual I-E genotype combination was found in 1.2% of circulating strains [24]. The combinations I2-E1 and I1-E2 were detected in 15.4% of RVA strains in India during 1990–2000 [28] and in 6.5% in Iran during 2021-2022 [29].

Multiple amino acid substitutions were detected in both VP6 and NSP4 genes. While many of these variants own key positions in the proteins, their functional impact remains unknown. VP6 protein is crucial as it is used in molecular and serological diagnostic tests for RVA due to its high conservation [30, 31]. The genetic analysis in this study showed that I2 was more conserved compared to I1 and I3, since only 16% of the I2 strains carried substitutions. This finding is in concordance with a similar study in South Africa, in which only I1 and I2 genotypes were described and I2 was found more conserved than I1 as it carried only two substitutions [31].

VP6 also contains four major antigenic regions [32]. Nyaga et al. described many substitutions in I1 antigenic region III, three of which (Y248F, V252I, and I253V) were also presented in our samples, but their functional effect is unknown [31]. Changes at the antigenic regions should be closely monitored since it could potentially affect the efficiency of the rotavirus detection methods and the future development of a VP6-based vaccine as it also induces the development of neutralization antibodies such as the capsid proteins VP7 and VP4 [33].

NSP4 is an essential protein for virus morphogenesis and pathogenesis. In the present study, nine possibly *novel* substitutions were found in the NSP4 gene. Most substitutions were detected in the VP4-binding domain, which also contains the toxic peptide and the interspecies variable domain (ISVD). According to other studies characterizing the nucleotide sequence of the NSP4 gene, the ISVD region shows great heterogenicity and the amino acids vary according to genotype [18, 34–37]. Limited functional studies exist and therefore the effects of these variants on the functionality and immunogenicity of the corresponding protein remain unknown.

Of interest are the substitutions in amino acid 131 in the region of the toxic peptide, in which the majority of the strains of this study carried the H131 and E2 strains mainly carried Y131. Ball et al. conducted a functional study for this amino acid on infant mice and they found that substitution in amino acid 131 has an effect on the enterotoxin properties of NSP4 [38]. Specifically, they reported that the Y131K substitutions resulted in the absence of diarrhoea. Studies from Brazil between 1990–2000 and 1987–2003 have reported that Y131 was detected only in E2 strains, while E1 strains had H131, and there were no data regarding E3 strains [39, 40]. Srivastava et al. showed that patients infected with a strain carrying Y131 experienced more severe diarrhoea [34]. Even though the severity of symptoms was not evaluated in the present study, statistical analysis showed that children infected with an unusual strain carrying the E2 genotype had a higher chance of exhibiting dehydration, which may indicate more severe diarrhoea. This result may also be related to the fact that Y131 was detected more in E2 strains.

Limitations of the present study included the moderate detection rates of both VP6 and NSP4 genes in RVA-positive fecal samples. However, similar detection rates have also been reported in other studies, possibly due to poor sample storage conditions or the presence of RNases resulting in fragmentation of the viral RNA genome, presence of PCR inhibitors, or inability of primers to hybridize [8, 41]. Another limitation of our study was that the analysis was based only on four genes (VP7, VP4, VP6, and NSP4) and not on the complete genotype constellation, which would provide more information about the genetic evolution of the strains. Future prospective studies are necessary to confirm our findings.

This is the first study of VP6 and NSP4 epidemiology and molecular characterization of unusual RVA strains in Greece, in which the unusual I3 and E3 genotypes, the reassortant I2-E3 human strains, and many substitutions in significant domains of NSP4 gene were detected. Furthermore, a significant clinical association between dehydration and E2 genotype was described.

Continuous surveillance of the distribution of RVA genotypes based on the whole genome, molecular characterization, and their association with epidemiological and clinical data is important for the better knowledge of the virus' evolution, disease prognosis, and upgrading RVA vaccines.

## 5. Conclusions

In this study, the genotype distribution of the VP6 and NSP4 genes in unusual rotavirus strains was described. The association between the RVA genotype and the severity of the symptoms needs to be further investigated. The application of next-generation sequencing to investigate genotypic combinations in the complete viral genome in combination with phylogenetic analysis will probably provide answers to the origin and evolutionary relationship of these strains.

## Figures and Tables

**Table 1:** Unusual G-P-I-E genotypes of group A rotaviruses circulating in Greece in a 15-year period (2007–2021).

Genotypes (Gx-Px-Ix-Ex)		*n*	%
Strains with G unusual genotype	G8-P[8]-I1-EUD	1	2.94
	G10-PUD-I2-E2	1	2.94

Strains with P unusual genotype	G3-P[9]-I2-E3	7	20.59
	G3-P[9]-I2-E2	2	5.89
	G9-P[10]-I1-E1	2	5.89
	G2-P[6]-I2-EUD	1	2.94
	G3-P[9]-I3-E3	1	2.94
	G4-P[6]-I2-E2	1	2.94
	G4-P[6]-I1-E1	1	2.94
	G4-P[9]-I1-E1	1	2.94
	G4-P[9]-I3-E3	1	2.94
	G9-P[9]-I2-E2	1	2.94
	G9-P[9]-I2-E3	1	2.94
	G12-P[6]-I1-E1	1	2.94
	G12-P[6]-I1-EUD	1	2.94
	G12-P[11]-I1-E2	1	2.94

Strains with G and P unusual genotypes	G8-P[14]-I2-E2	4	11.76
	G6-P[9]-I2-EUD	2	5.89
	G6-P[9]-I2-E2	1	2.94
	G6-P[9]-I2-E3	1	2.94
	G6-P[14]-I2-E2	1	2.94
	G8-P[14]-I2-EUD	1	2.94

Total		34	100

EUD = unidentified E genotype.

**Table 2:** Significant substitutions observed in the NSP4 gene.

E genotypes	Number of strains *n* (%)	Domain	Substitutions	Alleles
E2	1 (8.3)	H2	T78A	Homozygous
	2 (16.7)	VP4-binding domain	D140N	Homozygous (*n* = 1) and heterozygous (*n* = 1)

E3	1 (9.1)	H1	D19G	Heterozygous
	2 (18.2)	VP4-binding domain	K141R	Homozygous

## Data Availability

The data used to support the findings of this study are included within the article.

## Supplementary Materials

Supplementary Materials Supplementary Table 1: genotypes of study rotavirus group A (RVA) strains, collection year, and characteristics (demographic and clinical) of children infected with unusual RVA strains (*n* = 34). Abbreviations: N/A, not available; nt, nucleotide; UD, unidentified. Supplementary Table 2: demographic, clinical, and laboratory data of patients who were infected with a RVA strain carrying the I2 (*n* = 25) and non-I2 (*n* = 9) genotypes. *Notes.* Normal range for K^+^, N^+^, Ca^2+^, and Cl^−^ was 3.5–5.5 mmol/L, 135–150 mmol/L, 8.2–11.0 mg/dL, and 95–110 mmol/L, respectively. CRP normal range was 0-1 mg/L for children <1 year old and 1–10 mg/L for children 1–16 years old. Urea and creatinine normal ranges were 10–35 mg/dL and 0.2–1.0 mg/dL, respectively. Neutrophils and lymphocytes' normal ranges were 1.0–10.0 × 10^3^/mm^3^ and 1.5–17.0 × 10^3^/mm^3^ of total white blood cells, respectively. Abbreviations: NA, not applicable. Supplementary Table 3: demographic, clinical, and laboratory data of patients who were infected with a RVA strain carrying the E2 (*n* = 13) and E3 (*n* = 11) genotypes. *Notes.* Normal range for K^+^, N ^+^, Ca^2+^, and Cl^−^ were 3.5–5.5 mmol/L, 135–150 mmol/L, 8.2–11.0 mg/dL, and 95–110 mmol/L, respectively. CRP normal range was 0-1 mg/L for children of <1 year old and 1–10 mg/L for children 1–16 years old. Urea and creatinine normal ranges were 10–35 mg/dL and 0.2–1.0 mg/dL, respectively. Neutrophils and lymphocytes' normal ranges were 1.0–10.0 × 10^3^/mm^3^ and 1.5–17.0 × 10^3^/mm^3^ of total white blood cells, respectively. Statistically significant *p* values (<0.05) are marked in bold. Abbreviations: NA, not applicable.
