# Supplementary material for: Genotyping and Molecular Characterization of VP6 and NSP4 Genes of Unusual Rotavirus Group A Isolated from Children with Acute Gastroenteritis
Source: Adv Virol. 2024 Jul 4;2024:3263228. doi: 10.1155/2024/3263228 (PMC11239230; doi:10.1155/2024/3263228)
Supplement: Supplementary Materials — Supplementary Table 1: genotypes of study rotavirus group A (RVA) strains, collection year, and characteristics (demographic and clinical) of children infected with unusual RVA strains (n = 34). Abbreviations: N/A, not available; nt, nucleotide; UD, unidentified. Supplementary Table 2: demographic, clinical, and laboratory data of patients who were infected with a RVA strain carrying the I2 (n = 25) and non-I2 (n = 9) genotypes. Notes. Normal range for K+, N+, Ca2+, and Cl− was 3.5–5.5 mmol/L, 135–150 mmol/L, 8.2–11.0 mg/dL, and 95–110 mmol/L, respectively. CRP normal range was 0-1 mg/L for children <1 year old and 1–10 mg/L for children 1–16 years old. Urea and creatinine normal ranges were 10–35 mg/dL and 0.2–1.0 mg/dL, respectively. Neutrophils and lymphocytes' normal ranges were 1.0–10.0 × 103/mm3 and 1.5–17.0 × 103/mm3 of total white blood cells, respectively. Abbreviations: NA, not applicable. Supplementary Table 3: demographic, clinical, and laboratory data of patients who were infected with a RVA strain carrying the E2 (n = 13) and E3 (n = 11) genotypes. Notes. Normal range for K+, N +, Ca2+, and Cl− were 3.5–5.5 mmol/L, 135–150 mmol/L, 8.2–11.0 mg/dL, and 95–110 mmol/L, respectively. CRP normal range was 0-1 mg/L for children of <1 year old and 1–10 mg/L for children 1–16 years old. Urea and creatinine normal ranges were 10–35 mg/dL and 0.2–1.0 mg/dL, respectively. Neutrophils and lymphocytes' normal ranges were 1.0–10.0 × 103/mm3 and 1.5–17.0 × 103/mm3 of total white blood cells, respectively. Statistically significant p values (<0.05) are marked in bold. Abbreviations: NA, not applicable. [file 3263228.f1.zip › Supplementary Table 1 (2).docx]

**Supplementary Table 1.** Genotypes of study Rotavirus group A (RVA) strains, collection year and chararacteristics (demographic and clinical) of children infected with unusual RVA strains (n=34).

| Rotavirus Genotype | VP6  Accession number | NSP4  Accession number | Collection Year | Demographic data | | Clinical Symptoms | | | | Hospitalization Period  (days) | Vesikari severity score |
| --- | --- | --- | --- | --- | --- | --- | --- | --- | --- | --- | --- |
|  |  |  |  | **Sex** | **Age**  **(months)** | **Diarrhoea** | **Vomiting** | **Fever** | **Dehydration** |  |  |
| G8P[14]I2EUD | ON009342 | Ν/Α | 2007 | M | N/A | Yes | No | No | No | N/A | N/A |
| G3P[9]I2E2 | ON004913 | ON004914 | 2009 | M | 9.5 | Yes | Yes | Yes | Yes | 3 | 10 |
| G12P[6]I1E1 | OM972707 | ON156793 | 2011 | M | 0.7 | Yes | No | No | No | 14 | 8 |
| G12P[6]I1EUD | ΟΝ156796 | Ν/Α | 2011 | M | 0.4 | Yes | Yes | No | No | 11 | 7 |
| G8P[8]I1EUD | OM333186 | Ν/Α | 2011 | M | 14.7 | Yes | No | Yes | No | 7 | 11 |
| G6P[9]I2EUD | ΟΝ156797 | Ν/Α | 2011 | F | 51.7 | Yes | Yes | Yes | Yes | 2 | 9 |
| G6P[9]I2E2 | ON185611 | OM283126 | 2011 | M | 2.2 | Yes | Yes | Yes | No | 4 | 10 |
| G8P[14]I2E2 | OM287400 | OM283121 | 2012 | F | 77.1 | Yes | No | Yes | Yes | 2 | 8 |
| G4P[6]I2E2 | ON185612 | OM283122 | 2013 | M | 0.6 | Yes | Yes | Yes | No | 10 | 8 |
| G2P[6]I2EUD | ON185613 | Ν/Α | 2013 | F | 11.1 | Yes | Yes | No | Yes | 4 | 13 |
| G4P[6]I1E1 | ON185614 | ON156785 | 2014 | M | 33.0 | Yes | Yes | No | Yes | 3 | 12 |
| G3P[9]I2E3 | OM972710 | OM287398 | 2017 | M | 9.8 | Yes | Yes | No | No | 3 | 10 |
| G3P[9]I2E3 | OM461378 | OM948988 | 2017 | M | 92.8 | No | Yes | No | No | 1 | 9 |
| G9P[10]I1E1 | OM972708 | ON156789 | 2019 | F | 33.0 | Yes | Yes | Yes | Yes | 4 | 11 |
| G9P[9]I2E2 | OM461377 | ON156786 | 2019 | M | 24.6 | Yes | Yes | Yes | Yes | 4 | 12 |
| G10PUDI2E2 | OM333185 | ON156787 | 2019 | F | 1.8 | Yes | No | Yes | No | 4 | 10 |
| G12P[11]I2E2 | OM303088 | ON156788 | 2019 | M | 6.8 | Yes | Yes | Yes | Yes | 6 | 16 |
| G9P[10]I1E1 | OM323986 | ON156790 | 2019 | M | 4.2 | Yes | Yes | Yes | No | 3 | 16 |
| G4P[9]I3E3 | ON185615 | ON156792 | 2019 | M | 27.5 | Yes | No | No | No | N/A | N/A |
| G3P[9]I3E3 | ON185616 | ON156791 | 2019 | M | 33.5 | Yes | No | Yes | No | 1 | 10 |
| G3P[9]I2E2 | OM303085 | OM283123 | 2020 | M | 22.3 | Yes | Yes | Yes | Yes | 4 | 16 |
| G4P[9]I1E1 | ON185617 | OM287399 | 2020 | M | 10.4 | No | Yes | Yes | No | 2 | 7 |
| G8P[14]I2E2 | OM281959 | OM283125 | 2020 | F | 101.0 | Yes | Yes | Yes | No | 3 | 11 |
| G8P[14]I2E2 | OM972709 | ON564370 | 2020 | F | 44.1 | Yes | No | Yes | No | 4 | 14 |
| G3P[9]I2E3 | OM281957 | ON564371 | 2020 | F | 45.9 | Yes | Yes | Yes | No | 4 | 12 |
| G9P[9]I2E3 | OM281958 | OM283124 | 2020 | M | 44.5 | Yes | Yes | Yes | No | 2 | 11 |
| G3P[9]I2E3 | ON185618 | OM362404 | 2020 | M | 13.9 | Yes | No | Yes | No | 3 | 13 |
| G3P[9]I2E3 | ΟΝ206978 | OM948989 | 2020 | M | 18.7 | Yes | No | No | No | N/A | N/A |
| G3P[9]I2E3 | ΟΝ206980 | OM948990 | 2020 | F | 2.0 | Yes | No | Yes | No | 3 | 9 |
| G3P[9]I2E3 | ΟΝ206981 | OM948991 | 2021 | M | 54.6 | Yes | Yes | Yes | No | 2 | 14 |
| G6P[14]I2E2 | ON971933 | ON971935 | 2021 | M | N/A | Yes | No | No | No | N/A | N/A |
| G8P[14]I2E2 | ΟΝ206982 | OM281956 | 2021 | M | 66.6 | No | Yes | No | No | 7 | 14 |
| G6P[9]I2E3 | ΟΝ206983 | OM281953 | 2021 | M | 21.3 | Yes | Yes | Yes | No | 2 | 12 |
| G6P[9]I2EUD | ON971934 | N/A | 2021 | F | 6.2 | Yes | No | Yes | No | 3 | 7 |
| Median (IQR) |  |  |  |  | 20.0  (6.4-44.4) |  |  |  |  | 3.0 (2.3-4.0) |  |

Abbreviations: N/A=not available, nt: nucleotide, UD: unidentified.
