# Supplementary material for: Genotyping and Molecular Characterization of VP6 and NSP4 Genes of Unusual Rotavirus Group A Isolated from Children with Acute Gastroenteritis
Source: Adv Virol. 2024 Jul 4;2024:3263228. doi: 10.1155/2024/3263228 (PMC11239230; doi:10.1155/2024/3263228)
Supplement: Supplementary Materials — Supplementary Table 1: genotypes of study rotavirus group A (RVA) strains, collection year, and characteristics (demographic and clinical) of children infected with unusual RVA strains (n = 34). Abbreviations: N/A, not available; nt, nucleotide; UD, unidentified. Supplementary Table 2: demographic, clinical, and laboratory data of patients who were infected with a RVA strain carrying the I2 (n = 25) and non-I2 (n = 9) genotypes. Notes. Normal range for K+, N+, Ca2+, and Cl− was 3.5–5.5 mmol/L, 135–150 mmol/L, 8.2–11.0 mg/dL, and 95–110 mmol/L, respectively. CRP normal range was 0-1 mg/L for children <1 year old and 1–10 mg/L for children 1–16 years old. Urea and creatinine normal ranges were 10–35 mg/dL and 0.2–1.0 mg/dL, respectively. Neutrophils and lymphocytes' normal ranges were 1.0–10.0 × 103/mm3 and 1.5–17.0 × 103/mm3 of total white blood cells, respectively. Abbreviations: NA, not applicable. Supplementary Table 3: demographic, clinical, and laboratory data of patients who were infected with a RVA strain carrying the E2 (n = 13) and E3 (n = 11) genotypes. Notes. Normal range for K+, N +, Ca2+, and Cl− were 3.5–5.5 mmol/L, 135–150 mmol/L, 8.2–11.0 mg/dL, and 95–110 mmol/L, respectively. CRP normal range was 0-1 mg/L for children of <1 year old and 1–10 mg/L for children 1–16 years old. Urea and creatinine normal ranges were 10–35 mg/dL and 0.2–1.0 mg/dL, respectively. Neutrophils and lymphocytes' normal ranges were 1.0–10.0 × 103/mm3 and 1.5–17.0 × 103/mm3 of total white blood cells, respectively. Statistically significant p values (<0.05) are marked in bold. Abbreviations: NA, not applicable. [file 3263228.f1.zip › Supplementary Table 3 (1).docx]

**Supplementary Table 3.** Demographic, clinical and laboratory data of patients that were infected with a RVA strain carrying the E2 (n=13) and E3 (n=11) genotype.

| **Variables** | | **E Genotype** | | ***p*-value** |
| --- | --- | --- | --- | --- |
|  |  | **E2**  (n=13) | **E3**  (n=11) |  |
| **Gender** | Male | 8 (47.1%) | 9 (52.9%) | 0.386 |
|  | Female | 5 (71.4%) | 2 (28.6%) |  |
| **Age Groups** | < 1 year old | 6 (75.0%) | 2 (25.0%) | NA |
|  | 1 to 4 years old | 3 (30.0%) | 7 (70.0%) |  |
|  | 4 to 6 years old | 1 (50.0%) | 1 (50.0%) |  |
|  | 6< years old | 2 (66.7%) | 1 (33.3%) |  |
| **Seasonal Pattern** | Fall | 2 (20.0%) | 8 (80.0%) | NA |
|  | Winter | 3 (60.0%) | 2 (40.0%) |  |
|  | Spring | 6 (100.0%) | 0 (0.0%) |  |
|  | Summer | 2 (66.7%) | 1 (33.3%) |  |
| **Residence** | Urban | 10 (55.6%) | 8 (44.4%) | >0.999 |
|  | Rural | 3 (50.0%) | 3 (50.0%) |  |
| **Diarrhoea** | No | 1 (33.3%) | 2 (66.7%) | 0.553 |
|  | Yes | 11 (61.1%) | 7 (38.9%) |  |
| **Vomit** | No | 3 (50.0%) | 3 (50.0%) | >0.999 |
|  | Yes | 9 (60.0%) | 6 (40.0%) |  |
| **Fever** | No | 2 (50.0%) | 2 (50.0%) | >0.999 |
|  | Yes | 10 (58.8%) | 7 (41.2%) |  |
| **Dehydration** | No | 6 (40.0%) | 9 (60.0%) | **0.019** |
|  | Yes | 6(100.0%) | 0 (0.0%) |  |
| **Co-infection** | No | 11 (50.0%) | 11 (50.0%) | 0.482 |
|  | Yes | 2 (100%) | 0 (0.0%) |  |
| **Potassium (K^+^)** | Normal | 8 (44.4%) | 10 (55.6%) | 0.214 |
|  | Abnormal | 3 (100.0%) | 0 (0.0%) |  |
| **Sodium (Na^+^)** | Normal | 10 (55.6%) | 8 (44.4%) | 0.586 |
|  | Abnormal | 1 (33.3%) | 2 (66.7%) |  |
| **Calcium (Ca ^2+^)** | Normal | 9 (56.2%) | 7 (43.8%) | NA |
|  | Abnormal | 0 (NA) | 0 (NA) |  |
| **Chlorine (Cl^-^)** | Normal | 11 (55.0%) | 9 (45.0%) | 0.476 |
|  | Abnormal | 0 (0.0%) | 1 (100.0%) |  |
| **CRP** | Normal | 0 (NA) | 0 (NA) | NA |
|  | Abnormal | 7 (46.7%) | 8 (53.3%) |  |
| **Urea** | Normal | 10 (58.8%) | 7 (41.2%) | 0.311 |
|  | Abnormal | 1 (25.0%) | 3 (75.0%) |  |
| **Creatinine** | Normal | 10 (50.0%) | 10 (50.0%) | >0.999 |
|  | Abnormal | 1 (100.0%) | 0 (0.0%) |  |
| **Neutrophils** | Normal | 5 (45.5%) | 6 (54.5%) | >0.999 |
|  | Abnormal | 4 (50.0%) | 4 (50.0%) |  |
| **Lymphocytes** | Normal | 5 (71.4%) | 2 (28.6%) | 0.170 |
|  | Abnormal | 4 (33.3%) | 8 (66.6%) |  |

Notes: Normal range for K^+^, N ^+^, Ca^2+^, Cl^-^ were 3.5-5.5 mmol/L, 135-150 mmol/L, 8.2 -11.0 mg/dL and 95-110 mmol/L, respectively. CRP normal range was 0-1mg/L for children <1 year old and 1-10 mg/L for children 1-16 years old. Urea, creatinine normal ranges were 10-35 mg/dL and 0.2-1.0 mg/dL, respectively. Neutrophils and lymphocytes normal ranges were 1.0-10.0 x 10^3^ /mm^3^ and 1.5-17.0 x 10^3^/mm^3^of total white blood cells, respectively. Statistically significant p-values (<0.05) are marked in bold. Abbreviations: NA=not applicable
